# Analysis of risk factors for hypothyroidism in initially euthyroid patients with positive thyroid autoantibodies: a multicenter retrospective cohort study

**DOI:** 10.3389/fendo.2026.1827517

**Published:** 2026-05-18

**Authors:** Xue Feng, Xuan Lyu, Yating Gao, Xu Gao, Rui Gao

**Affiliations:** 1 Xiyuan Hospital, China Academy of Chinese Medical Sciences, Beijing, China; 2 School of Public Health, Peking University, Beijing, China; 3 Beijing University of Chinese Medicine, Beijing, China; 4 Key Laboratory of Epidemiology of Major Diseases (Peking University), Ministry of Education, Beijing, China; 5 Peking University Institute of Environmental Medicine, Beijing, China; 6 Center for Healthy Aging, Peking University Health Science Center, Beijing, China

**Keywords:** cohort study, Hashimoto’s thyroiditis, hypothyroidism, risk factors, thyroid peroxidase antibody

## Abstract

**Background:**

Thyroid autoantibody positivity is a common immune marker and a major precursor of hypothyroidism. Currently, there is a lack of effective tools and risk stratification criteria for identifying individuals at risk of its progression to either overt or subclinical hypothyroidism.

**Objective:**

To investigate the independent risk factors for future onset of hypothyroidism and determine an optimal anti-thyroid peroxidase antibody (TPOAb) cutoff for identifying high-risk individuals.

**Methods:**

A multicenter retrospective cohort study was conducted. A total of 669 patients initially diagnosed with positive thyroid autoantibodies with positive thyroid autoantibodies (TPOAb and/or TgAb) in Nanjing from January 2018 to December 2024 were enrolled. Baseline demographic data, thyroid function parameters, and autoantibody levels were collected. The primary endpoint was progression to overt or subclinical hypothyroidism during follow-up. Cox proportional hazards regression models were used to identify independent risk factors. The optimal risk-stratification cutoff value for TPOAb was determined using the maximum selection method. Risk stratification performance was evaluated using the Akaike Information Criterion (AIC) and the concordance index (C-index).

**Results:**

During a median follow-up of 3.0 years, 155 patients progressed to overt or subclinical hypothyroidism. Multivariate Cox regression analysis revealed that age was a protective factor (18–45 years vs ≤18 years: HR = 0.36, 95% CI: 0.22–0.58, *P* < 0.001; ≥45 years vs ≤18 years: HR = 0.45, 95% CI: 0.26–0.80, *P* = 0.007). Baseline thyroid-stimulating hormone (TSH), free thyroxine (FT4), and TPOAb levels were identified as independent risk factors (all *P* < 0.05). The optimal risk-stratification cutoff for TPOAb was 187 IU/mL. Risk stratification using this cutoff demonstrated a lower AIC value (811.05) and a higher C-index (0.700) compared to the model using the commonly used clinical cutoff of 60 IU/mL.

**Conclusion:**

This study identifies that age, baseline TSH, FT4, and TPOAb levels are independent risk factors for the progression to hypothyroidism in patients with hypothyroidism onset in initially euthyroid patients with positive thyroid autoantibodies. A TPOAb cutoff of 187 IU/mL provides better risk discrimination and can be utilized for clinical identify high-risk patients and individualized monitoring.

## 1 Introduction

Thyroid autoantibody positivity is a common precursor of hypothyroidism characterized by lymphocytic infiltration of the thyroid gland and progressive destruction of thyroid tissue. It represents the most common cause of hypothyroidism (1). Epidemiological studies indicate a rising trend in both the incidence and prevalence of thyroid autoantibody positivity in recent years. The positive rate of thyroid autoantibodies in the Chinese population is approximately 14.19% (2). Individuals with positive thyroid autoantibodies exhibit a significant gender disparity, with an occurrence rate in females 15–20 times higher than in males (3); women aged 30–50 years constitute a high-risk group (4). Notably, a subset of euthyroid individuals with positive thyroid autoantibodies continues to experience numerous non-specific symptoms, such as fatigue, mood disorders, goiter, and diminished quality of life, even during the euthyroid phase or after hypothyroidism has been corrected with hormone replacement therapy. This phenomenon is believed to be closely associated with a persistent autoimmune inflammatory state within the thyroid (5–8).

The clinical course of thyroid autoimmunity is protracted and characterized by notable individual heterogeneity. Although the ultimate outcome is often thyroid failure, the time interval from diagnosis to the development of hypothyroidism varies widely among patients. Currently, there is a lack of effective therapeutic strategies to halt the progression of autoimmune thyroid damage to overt hypothyroidism. In this context, identifying high-risk individuals prone with positive thyroid autoantibodies prone to hypothyroidism and implementing risk-stratified management have become crucial for optimizing clinical follow-up strategies and exploring windows for early intervention. However, the prevention and management of autoimmune-related thyroid dysfunction still face several core challenges: the optimal timing for early intervention remains unclear; objective methods and unified criteria for identifying high-risk individuals for disease progression are lacking; and evidence-based effective early-intervention drugs, along with corresponding efficacy evaluation systems are still absent. These bottlenecks not only hinder the implementation of precise clinical management but also impede the development and evaluation of related therapeutic strategies.

Traditionally, age, baseline thyroid function, and anti-thyroid peroxidase antibody (TPOAb) titer have been considered potential risk indicators (9). However, the current clinical cut-off value for TPOAb is primarily established for diagnostic purposes, and its efficacy and optimal threshold for stratifying the risk of long-term functional outcomes have not been clearly defined. Furthermore, the protective effect of age and the clinical significance of thyroid-stimulating hormone (TSH) levels within the upper normal range require quantitative validation in prospective cohorts. Systematically elucidating the independent risk-association of these factors will provide a theoretical basis for constructing a concise and practical clinical risk assessment and decision-making.

This study aims to systematically evaluate multi-dimensional indicators, including demographic characteristics, baseline thyroid function, and autoantibody profiles, through a retrospective cohort study to identify independent risk factors for hypothyroidism in initially euthyroid patients with positive thyroid autoantibodies. We focus on quantifying the protective effect of age and determining the optimal TPOAb threshold for risk stratification. The goal is to identify independent risk factors and determine an optimal TPOAb cutoff for risk stratification to provide a scientific basis for the individualized management and early monitoring of patients with positive thyroid autoantibodies, as well as for the formulation of enrollment criteria for future intervention studies.

## 2 Materials and methods

### 2.1 Study design and data source

This study was a multicenter retrospective cohort study designed to investigate the risk factors for progression to overt or subclinical hypothyroidism in initially euthyroid patients with positive thyroid autoantibodies. The data were sourced from the electronic medical record systems of all 315 hospitals in Nanjing City from January 2018 to December 2024. The study protocol was approved by the Ethics Review Committee of Ethics Committee of Xiyuan Hospital, China Academy of Chinese Medical Sciences (Approval No.: 2025XLA033-1). Informed consent was waived due to the use of previously anonymized data.

### 2.2 Study population

The study enrolled initially euthyroid patients with positive thyroid autoantibodies, defined as positivity for serum TPOAb and thyroglobulin antibodies (TgAb), or positivity for a single antibody (TPOAb or TgAb) (9).

Inclusion criteria were: (1) positive for TPOAb and/or TgAb at baseline; (2) euthyroid status at the time of initial diagnosis; (3) at least two follow-up records; and (4) a follow-up duration of at least 2 years.

Exclusion criteria included: (1) a baseline diagnosis of overt or subclinical hypothyroidism; (2) concomitant other thyroid diseases (e.g., Graves’ disease, and confirmed or suspected neoplastic thyroid lesions such as thyroid carcinomas or follicular adenomas); (3) patients with severe systemic diseases; and (4) those with missing follow-up data or loss to follow-up. Of note, patients presenting with benign diffuse or nodular goiters, which are common structural manifestations of the underlying autoimmune process in Hashimoto’s disease, were not considered as having thyroid tumors and were therefore not excluded from the study cohort.

### 2.3 Data collection and definitions

All data were extracted from the hospital electronic medical record systems, laboratory information systems, and outpatient follow-up records managed by the Nanjing Municipal Health Commission. Baseline characteristics collected at the first hospitalization included demographic data (age, sex) and the following thyroid function and antibody indices: thyroid-stimulating hormone (TSH), free triiodothyronine (FT3), free thyroxine (FT4), TPOAb, and TgAb. It should be noted that TPOAb and TgAb testing in these initially euthyroid patients was not performed as routine mass screening. Rather, testing was prompted by specific clinical indications, including physical examination findings (e.g., palpable goiter or thyroid nodules), incidental ultrasound abnormalities suggestive of thyroiditis (e.g., hypoechogenicity or heterogeneous texture), a significant family history of autoimmune thyroid disease, or other non-specific symptoms raising suspicion of an underlying autoimmune state. In compliance with institutional data protection and patient privacy regulations, exact dates of birth and continuous age data were de-identified during extraction. For analysis, age was therefore provided and categorized into three groups: ≤18 years (adolescent), 18–45 years (young adult), and ≥45 years (middle-aged and elderly). The primary outcome was defined as progression to either overt hypothyroidism or subclinical hypothyroidism. Overt hypothyroidism was defined as TSH > 4.20 mIU/L and FT4 < 12 pmol/L, while subclinical hypothyroidism was defined as TSH > 4.20 mIU/L with FT4 within the normal range. Follow-up time was calculated from the date of the patient’s first visit. The endpoint event was the occurrence of overt or subclinical hypothyroidism or the date of the last follow-up, whichever occurred first.

### 2.4 Statistical analysis

Statistical analyses were performed using R software (version 4.4.1). Continuous variables were presented as mean ± standard deviation for normally distributed data and compared using the t-test. Non-normally distributed variables were described as median (interquartile range [IQR]) and compared using the Mann-Whitney U test. Categorical data were presented as frequency (percentage) and compared using the χ² test or Fisher’s exact test.

To handle missing values in baseline continuous variables (TPOAb, TgAb, FT3), multiple imputation was performed using the Random Forest method, generating 10 complete datasets. All subsequent analyses were based on the pooled estimates from these imputed datasets. First, univariate Cox proportional hazards regression analyses were conducted for each baseline variable to calculate the hazard ratios (HRs) and 95% confidence intervals (CIs). Demographic variables and variables with a *P*-value < 0.05 in the univariate analysis were included in a multivariate Cox proportional hazards model. Forward stepwise regression was used to identify independent risk factors for the progression of hypothyroidism onset.

After confirming TPOAb as an independent risk factor, its optimal risk-stratification cutoff value was further explored. To validate the robustness of this cutoff against different missing data handling strategies, a sensitivity analysis was performed. Specifically, the optimal cutoff was calculated and compared across datasets prepared (1) via complete-case analysis (excluding original missing TPOAb values), (2) median imputation, (3) random forest imputation, and (4) k-nearest neighbors (k-NN) imputation. To guarantee that the clinical threshold was derived strictly from true biological measurements rather than algorithmically imputed data, the complete-case approach was selected as the final analytical standard. The ‘surv_cutpoint’ function from the ‘survminer’ package was used on this complete-case subset to determine the optimal cutoff value of TPOAb for identifying patients at high risk the outcome event based on the maximum selection method. To evaluate the clinical value of this optimal cutoff, it was compared with a commonly used clinical cutoff (60 IU/mL): TPOAb as a binary variable defined by each cutoff was separately incorporated into Cox models, and the discriminative ability of risk stratification was evaluated using the AIC and the C-index.

Finally, based on the determined optimal cutoff, the study population was divided into groups. Kaplan-Meier survival curves were plotted, and the Log-rank test was used to compare differences in hypothyroidism-free survival between groups. The results of the multivariate Cox regression analysis were visualized in a forest plot. All statistical tests were two-sided, and a P value of < 0.05 was considered statistically significant.

## 3 Results

### 3.1 Baseline characteristics of the study population

**Figure 1:**
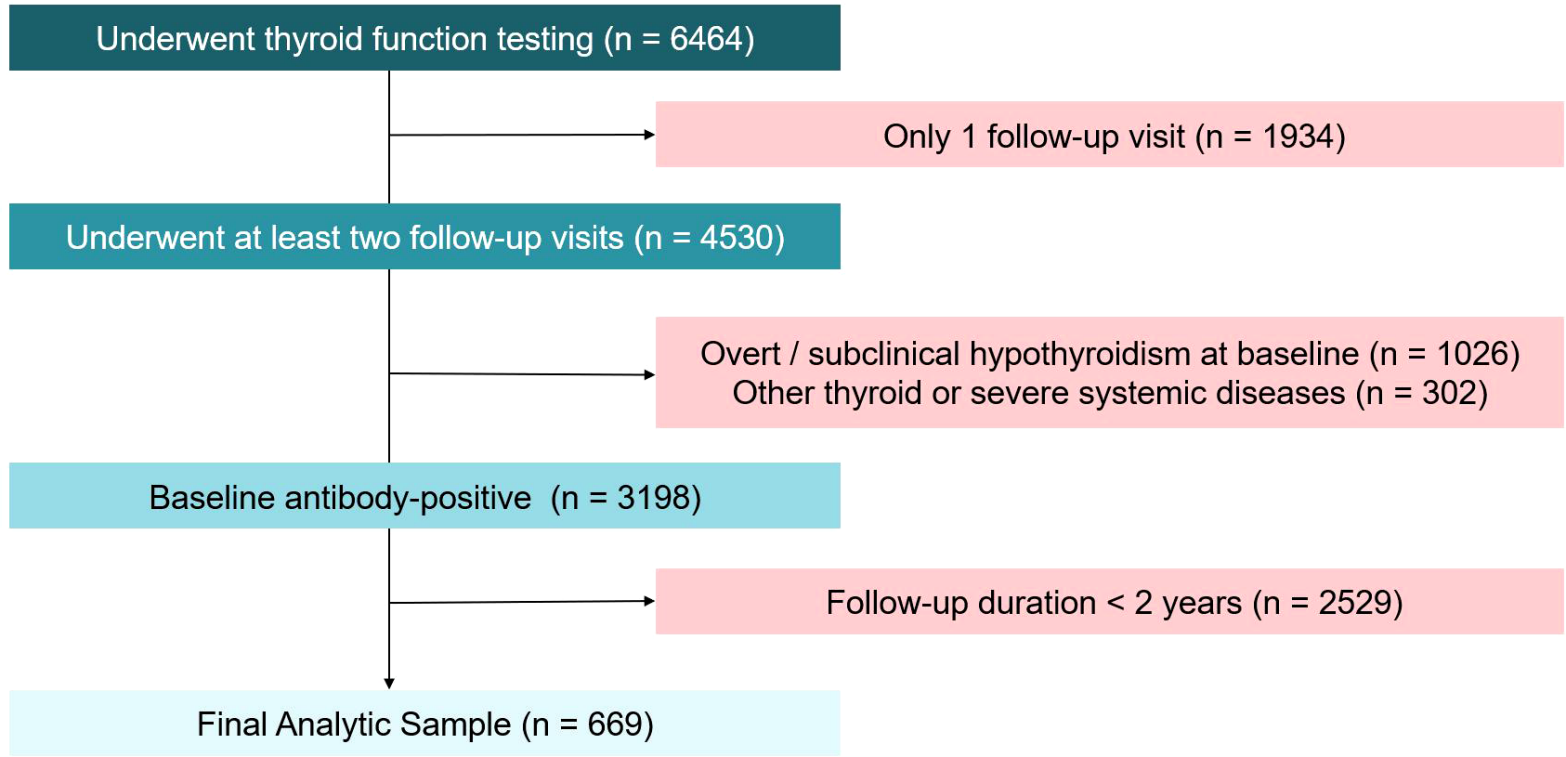
Flowchart of the study population selection process.

As illustrated in the patient selection flowchart (**Figure 1**), a total of 6464 individuals who underwent thyroid function testing were initially screened. After excluding 1934 patients with only one follow-up visit, 1026 patients with subclinical or overt hypothyroidism at baseline and 302 patients with other thyroid or severe systemic diseases, 3198 baseline antibody-positive patients remained. Subsequently, 2529 were excluded due to a follow-up duration of less than 2 years. This rigorous screening yielded a final analytic sample of 669 strictly euthyroid subjects. Prior to data imputation for the multivariable analysis, the original dataset contained missing values for certain baseline parameters: TPOAb in 271 cases (40.5%), TgAb in 216 cases (32.3%), and FT3 in 95 cases (14.2%). The median follow-up period for the entire study cohort was 3.0 years (interquartile range [IQR]: 2.3–4.0 years; range: 2.0–6.3 years). During this period, 155 patients developed the primary endpoint of overt or subclinical hypothyroidism.

Among the 669 subjects, the mean age was 34 years, with 51 (7.6%) male and 618 (92.4%) female (**Table 1**). Notably, the age distribution shifted across different stages of the cohort selection. During the exclusion phase, a substantial proportion of older individuals were filtered out due to pre-existing baseline hypothyroidism (e.g., 541 patients aged 18–45 years and 359 patients aged ≥45 years) (**Supplementary Table 1**). Consequently, within the final analytic sample, the age distribution was: 55 subjects (8.2%) were aged ≤18 years, 490 (73.2%) were aged 18–45 years, and 124 (18.5%) were aged ≥45 years. The baseline TPOAb level was 157.00 IU/mL [IQR: 34.77, 298.77], baseline TSH was 2.07 mIU/L [IQR: 1.31, 2.98], and baseline FT4 was 16.28 pmol/L [IQR: 14.30, 18.30].

**Table 1:** Comparison of baseline characteristics between the two groups.

Variable	Total (n = 669)	No progression (n = 514)^a^	Progression (n = 155)^b^	Test statistic^c^	P value
Sex				χ² = 0.056	0.813
male	51 (7.6%)	38 (7.4%)	13 (8.4%)		
female	618 (92.4%)	476 (92.6%)	142 (91.6%)		
Age group				χ² = 14.47	<0.001
≤18 years	55 (8.2%)	31 (6%)	24 (15.5%)		
18-45 years	490 (73.2%)	388 (75.5%)	102 (65.8%)		
≥45 years	124 (18.5%)	95 (18.5%)	29 (18.7%)		
Baseline TPOAb	157.00 [34.77, 298.77]	132.50 [30.87, 280.80]	236.00 [89.98, 411.67]	W = 9800	0.003
Baseline TGAb	284.90 [83.40, 500.00]	279.00 [80.21, 495.40]	299.00 [101.00, 556.70]	W = 15807	0.407
Baseline TSH	2.07 [1.31, 2.98]	1.99 [1.29, 2.83]	2.61 [1.54, 3.38]	W = 31948	<0.001
Baseline FT3	4.72 [4.32, 5.16]	4.73 [4.36, 5.13]	4.69 [4.20, 5.17]	W = 28990	0.714
Baseline FT4	16.28 [14.30, 18.30]	16.30 [14.60, 18.30]	16.10 [13.75, 18.27]	W = 41006	0.579

a No progression to clinical/subclinical hypothyroidism, b progression to clinical/subclinical hypothyroidism. c Chi-square test was used for categorical variables (Sex, Age group), and Wilcoxon rank-sum test was used for continuous variables (Baseline TPOAb, TGAb, TSH, FT3, FT4).

Comparison between groups based on study outcome (progression/non-progression) revealed significant differences between the progression group (n = 155) and the non-progression group (n = 514) in age categories (P < 0.001), baseline TPOAb level (P = 0.003), and baseline TSH level (P < 0.001). Specifically, the proportion of subjects in the ≤18-year age group was higher in the progression group (15.5% vs. 6.0%), and baseline TPOAb (236.00 vs. 132.50 IU/mL) and TSH (2.61 vs. 1.99 mIU/L) levels were significantly higher in the progression group compared to the non-progression group.

### 3.2 Results of univariate cox regression analysis

The results of the univariate Cox regression analysis are presented in **Table 2**. The analysis indicated that baseline TSH (HR = 1.25, 95%CI: 1.09–1.43, P < 0.001), baseline FT4 (HR = 1.02, 95% CI: 1.01–1.04, P = 0.011), and baseline TPOAb (HR = 1.14, 95% CI: 1.05–1.24, P = 0.003) levels were significantly and positively associated with the risk of the study outcome. No significant associations were found between the outcome risk and gender, baseline FT3, or baseline TgAb levels.

**Table 2:** Univariate cox regression analysis of progression to hypothyroidism in initially euthyroid patients with positive thyroid autoantibodies.

Variable	HR (95% CI)	P-value
Sex (female vs male)	0.83 (0.47–1.47)	0.52
Baseline TSH (per 1 mIU/L increase)	1.25 (1.09–1.43)	< 0.001
Baseline FT3 (per 1 pmol/L increase)	1.07 (0.91–1.26)	0.39
Baseline FT4 (per 1 pmol/L increase)	1.02 (1.01–1.04)	0.011
Baseline TPOAb (per 100 IU/mL increase)	1.14 (1.05–1.24)	0.003
Baseline TGAb (per 100 IU/mL increase)	1.02 (1.00–1.04)	0.074

### 3.3 Results of multivariate cox regression analysis

**Figure 2:**
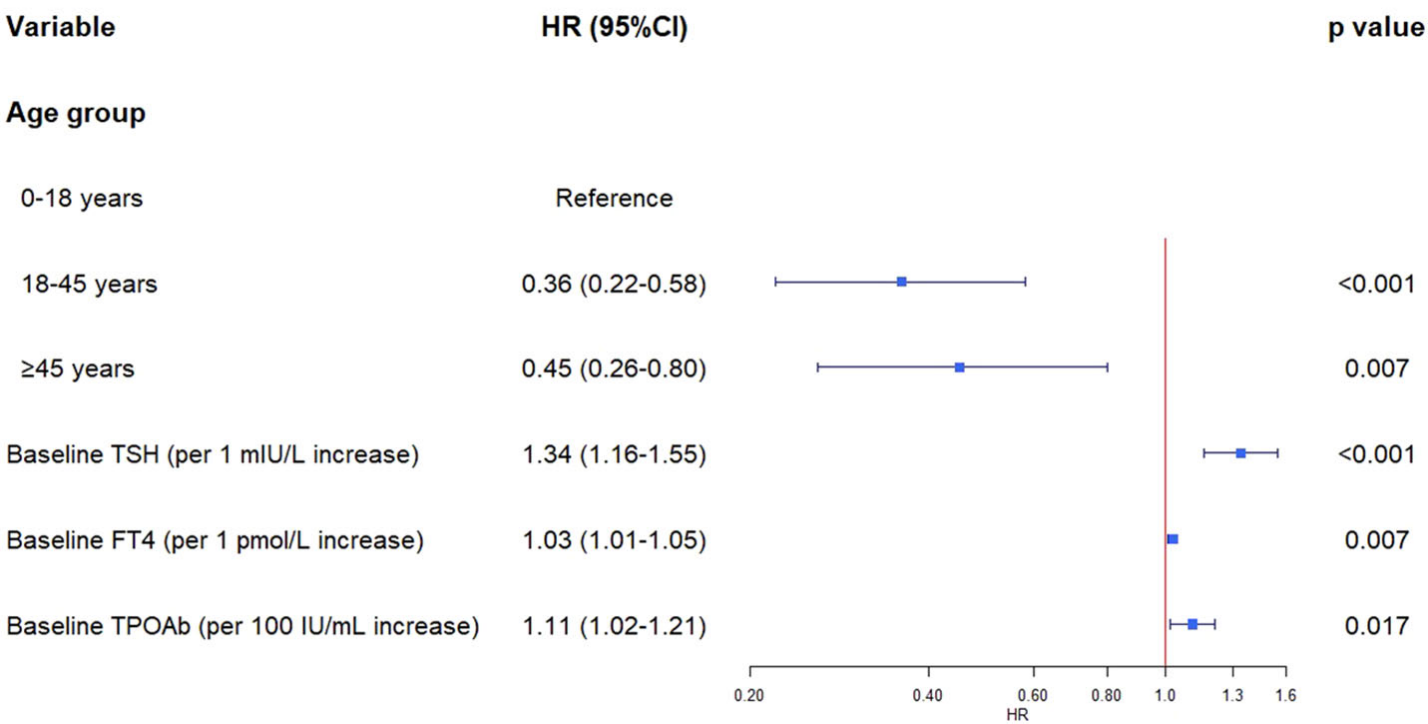
Forest plot of multivariate cox regression for progression to hypothyroidism in initially euthyroid patients with positive thyroid autoantibodies.

Variables that were statistically significant in the univariate analysis (age group, baseline TSH, FT4, TPOAb) were subsequently included in a multivariate Cox regression model. The results are displayed as a forest plot (**Figure 2**). There was no significant multicollinearity among these variables (all VIF values < 1.34).

After adjusting for other factors, age group, baseline TSH, FT4, and TPOAb levels remained independent risk factors associated with disease progression. Specifically, compared to the ≤18-year age group, the risk was reduced by 64% in the 18–45-year group (HR = 0.36, 95% CI: 0.22–0.58, P < 0.001) and by 55% in the ≥45-year group (HR = 0.45, 95% CI: 0.26–0.80, P = 0.007). For every 100 IU/mL increase in baseline TPOAb, the risk increased by 11% (HR = 1.11, 95% CI: 1.02–1.21, P = 0.017).

### 3.4 Determination and comparison of the optimal TPOAb cut-off point

After confirming TPOAb as an independent risk factor through multivariate Cox regression analysis, the optimal cut-off point for converting the continuous variable TPOAb into a categorical variable was further explored. The sensitivity analysis evaluating the impact of missing data handling strategies revealed highly consistent optimal cut-off values across the complete-case analysis (187.0 IU/mL) and advanced imputation algorithms (185.1 IU/mL for random forest and 187.9 IU/mL for k-NN) (**Supplementary Table 2**). Based on the predefined methodological criteria, the complete-case approach was selected as the final standard (n = 398). Using the surv_cutpoint method on this subset, the optimal TPOAb threshold for risk assessment of the study outcome was determined to be 187 IU/mL (maximum log-rank statistic = 3.61).

To evaluate the clinical value of this cut-off point, the stratification performance of the commonly used clinical cut-off point (60 IU/mL) was compared with that of the optimal cut-off point (187 IU/mL) (**Table 3**). When using the optimal cut-off point, the high TPOAb group (>187 IU/mL) had a higher HR = 2.02 (95% CI: 1.26–3.23) compared to the HR = 1.66 (95% CI: 0.96–2.86) obtained using the clinical cut-off point. Furthermore, the model constructed using the general clinical cut-off point was not statistically significant (P = 0.068).

**Table 3:** Comparison of different TPOAb cut-off stratification methods.

Model	HR (95% CI)	P value	AIC	C-index	Proportion of High TPOAb
TPOAb > 60 IU/mL (Clinical cut-off)	1.66 (0.96–2.86)	0.068	816.17	0.68	0.68
TPOAb > 187 IU/mL (Optimal cut-off)	2.02 (1.26–3.23)	0.004	811.05	0.7	0.44

Based on the sensitivity analysis, the cut-off evaluations presented in this table were conducted strictly on the complete-case subset (n = 398) with directly measured baseline TPOAb values. The 271 cases with missing original TPOAb data were excluded from this specific sub-analysis.

In terms of risk stratification performance, the optimal cut-off point model outperformed the clinical cut-off point model in both the Akaike Information Criterion (AIC: 811.05 vs. 816.17) and the concordance index (C-index: 0.700 vs. 0.684), indicating better discriminative capacity of the risk categories.

### 3.5 Survival analysis stratified by the optimal TPOAb cut-off point

**Figure 3:**
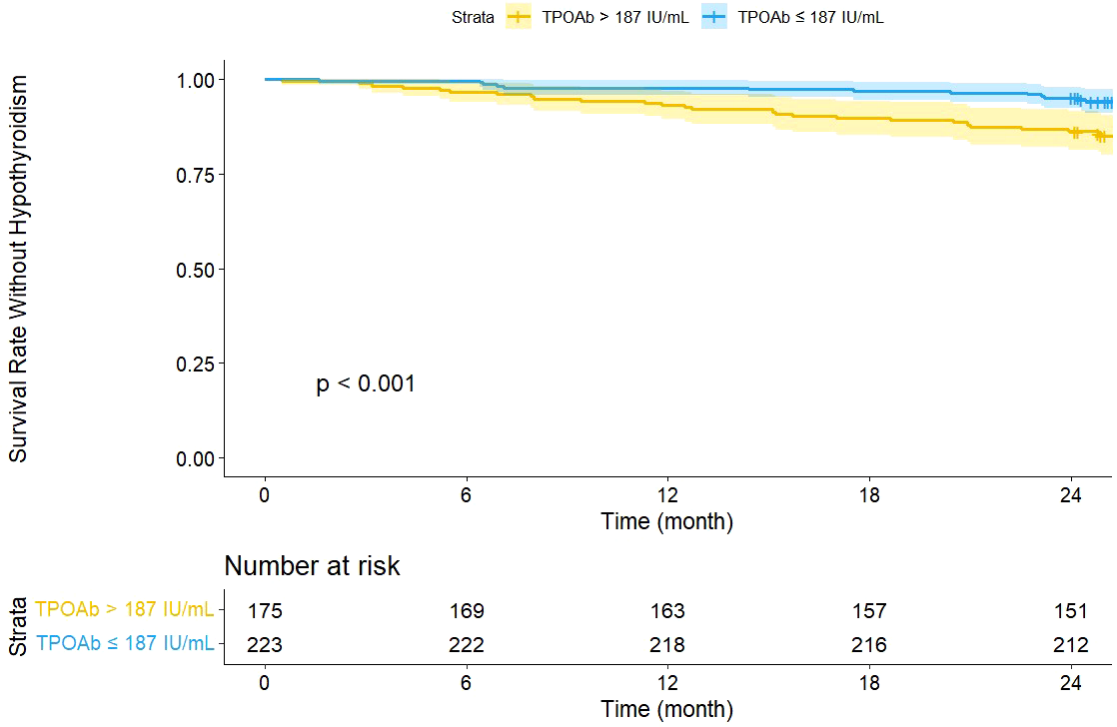
Survival curves grouped by optimal TPOAb cut-off.

Based on the determined optimal cut-off point (187 IU/mL), the study population was divided into a high TPOAb group (>187 IU/mL, n = 175) and a low TPOAb group (≤187 IU/mL, n = 223). Kaplan-Meier survival curves (**Figure 3**) demonstrated a significant difference in hypothyroidism-free survival rates between the two groups (Log-rank test, *P* < 0.001).

Throughout the 24-month follow-up period, the survival rate was significantly lower in the high TPOAb group compared to the low TPOAb group. The number at risk table showed that at the 24-month follow-up, 151 patients (86.3%) in the high TPOAb group and 212 patients (95.1%) in the low TPOAb group remained in the follow-up cohort and had not yet reached the endpoint. These results further confirm the importance of TPOAb as an independent risk factor for the study outcome.

## 4 Discussion

This study systematically evaluated the risk factors for progression to overt or subclinical hypothyroidism in 669 patients with initially euthyroid patients with positive thyroid autoantibodies. The main findings are as follows: first, age was the most significant protective factor in the multivariate analysis, with adult patients (especially those aged 18-45 years) demonstrating a significantly lower risk of progression compared to adolescent patients (≤18 years). Second, baseline thyroid function indices (TSH, FT4) were independent risk factors. Third, baseline TPOAb level was an independent risk factor in the multivariate model. Finally, we determined the optimal cut-off value for TPOAb in risk assessment (187 IU/mL), which outperformed the traditional clinical cut-off in identifying high-risk individuals. These findings provide important evidence-based support for risk stratification and individualized management of patients with patients with positive thyroid autoantibodies.

This study found that older age was associated with a significantly reduced risk of progression in the multivariate analysis. Compared to adolescents ≤18 years, patients aged 18-45 years had a 64% reduced risk of progression (HR = 0.36, P < 0.001), and patients ≥45 years had a 55% reduced risk (HR = 0.45, P = 0.007). Only a limited number of studies have investigated the natural course and outcomes of thyroid autoimmunity in children and adolescents (10–12). Of the 55 adolescent patients in our cohort, only 31 (56.4%) remained free from progression to overt or subclinical hypothyroidism during the two-year follow-up. This finding indicates a progression-free survival rate lower than that reported in previous studies (13, 14). Several mechanisms may underpin this observation. First, during puberty, the immune system undergoes functional remodeling under the regulation of sex hormones. These immunoendocrine changes may affect the regulation of autoimmune responses, thereby contributing to the progression of thyroid autoimmunity to some extent (15). Second, metabolic and endocrine changes associated with growth and development during puberty may increase the demand for thyroid hormone, rendering a thyroid gland with compromised functional reserve more prone to decompensation and the development of hypothyroidism (16). Third, adolescent patients may experience delayed diagnosis and treatment due to atypical symptoms, leading to a longer period without intervention (17, 18). However, the observed lower risk among older adults must be interpreted with caution due to the survival bias inherent in our study design. Because we strictly excluded patients with baseline hypothyroidism, individuals who rapidly progressed at a younger age were systematically filtered out. Therefore, the older adults in our cohort likely represent a “survivor” phenotype with a more indolent autoimmune process or robust compensatory reserve. This finding has important clinical implications, emphasizing the need for closer monitoring and more proactive intervention strategies for adolescent patients with positive thyroid autoantibodies. Clinicians should recognize age as a significant moderator of disease progression and implement more frequent thyroid function testing and a lower intervention threshold for this population.

Baseline TSH was the risk factor with the strongest effect in the multivariate model. For each 1 mIU/L increase in TSH, the risk of progression increased by 34% (HR = 1.34, P < 0.001). This finding is consistent with the natural history of hypothyroidism. An elevated TSH reflects the compensatory response of the hypothalamic-pituitary axis to insufficient thyroid hormone and is a sensitive indicator of thyroid function impairment (19). Notably, even within the normal reference range, higher TSH levels were associated with an increased risk of progression, supporting the view of TSH as a risk indicator for hypothyroidism in antibody-positive individuals (20). For each 1 pmol/L increase in baseline FT4, the risk of progression increased by 3% (HR = 1.03, P = 0.007). Although modest, this effect was statistically significant. This may seem counterintuitive, as decreased FT4 is typically associated with hypothyroidism. However, during the euthyroid stage, a mildly elevated FT4 may reflect a state of thyroid stress and compensatory enhancement of reserve function, serving as an early marker of thyroid dysfunction (19). It is noteworthy that FT3 did not show a significant association in the multivariate analysis. This may be because thyroid function exhibits significant age-dependence during childhood and adolescence. FT3 exhibits considerable physiological fluctuations throughout childhood and puberty which are associated with pubertal developmental stages (20), potentially confounding its independent indicator for risk assessment. Furthermore, during the development of primary hypothyroidism, serum T3/FT3 often remains within the reference range for a prolonged period, typically decreasing below the reference range only at a later stage. This suggests that abnormal FT3 is more likely to reflect a later stage of failed compensation, thereby limiting its utility in risk assessment for early progression in patients with positive thyroid autoantibodies (21).

Baseline TPOAb level was confirmed as an independent risk factor for progression. For each 100 IU/mL increase in TPOAb, the risk of progression increased by 11% (P = 0.017). The presence of TPOAb has long served as a primary basis and reference for the diagnosis and prognosis of autoimmune thyroid conditions (20, 22, 23). The optimal TPOAb cut-off value of 187 IU/mL identified in this study holds significant clinical importance. Compared to the commonly used clinical cut-off (60 IU/mL, with slight variations among different laboratories), the optimal cut-off demonstrated better stratification performance: a lower AIC (811.05 vs. 816.17), a higher C-index (0.700 vs. 0.684), and a higher HR (2.02 vs. 1.66). This finding suggests that the antibody cut-off used for diagnosis may not be optimal for risk assessment of disease progression, and cut-off selection needs to be optimized for specific clinical endpoints (22). Although cut−off values reported in the literature vary due to differences in assays, populations, and follow−up durations, the threshold identified here is higher than those found in some prior studies (24, 25). Notably, using the optimal cut-off classified only 44.0% of patients as high-risk, whereas the clinical cut-off classified 67.8%. This indicates that the optimal cut-off can more specifically identify truly high-risk patients, avoiding unnecessary intervention in low-risk patients. However, this C-index was derived from the same dataset without internal validation (e.g., bootstrapping) and should not be interpreted as a measure of predictive accuracy for an external population. In contrast, TgAb exhibited no significant risk indication in the multivariate analysis (HR = 1.02, P = 0.074), which is also consistent with previous research findings (26). While our findings demonstrate that the optimal baseline TPOAb cut-off (187 IU/mL) can effectively stratify the risk of progression to hypothyroidism, this finding should not be misinterpreted as a recommendation for universal TPOAb screening in the general asymptomatic euthyroid population. Rather, this risk-stratification strategy is intended specifically for patients who already present with clinical indications for thyroid evaluation (e.g., goiter, sonographic abnormalities, or a strong family history). For this targeted population, our proposed cut-off provides clinicians with a practical and evidence-based tool to identify high-risk individuals who warrant more frequent monitoring and proactive management.

This study also has several limitations. First, the sample included only patients from Nanjing, with an excessively high proportion of females (92.4%). Although previous studies have similarly found that the occurrence rate in females is approximately ten times that in males (27), this may limit the generalizability of the results to other regions and male patients. Second, despite adjusting for major clinical variables, some potential confounding factors, such as iodine intake (28), selenium status (29), and genetic and environmental factors (30), which may influence disease progression, were not considered. Finally, the follow-up period was relatively short. A longer follow-up is needed to confirm the long-term value of these risk factors. Finally, the relatively short follow-up period (median 3.0 years) may underestimate the true long-term cumulative incidence of hypothyroidism, as autoimmune thyroid destruction is typically a slow process spanning decades. Consequently, our identified TPOAb cut-off primarily reflects short- to medium-term risk. Future research should focus on the following aspects: First, validating the risk-stratification performance of the optimal TPOAb cut-off in more representative populations in terms of sex and age distribution. Second, investigating whether dynamic monitoring of TPOAb changes has better clinical value than a single measurement (31, 32). Third, developing integrated risk stratification approaches combining thyroid ultrasound features (e.g., hypoechogenicity, heterogeneous texture) and serum markers (33, 34). Finally, exploring novel biomarkers, such as thyroid-stimulating antibody subtypes or cytokine profiles, to further improve risk assessment accuracy (35).

In conclusion, this study demonstrates that age, baseline TSH, FT4, and TPOAb levels are independent risk factors for the progression of hypothyroidism in initially euthyroid patients with positive thyroid autoantibodies. Among these, age is a significant protective factor, while higher TSH and TPOAb levels are risk factors. The optimal TPOAb cut-off value determined (187 IU/mL) improved the identification of high-risk individuals. These findings provide an evidence-based foundation for risk stratification in patients with positive thyroid autoantibodies. It is suggested that patients with TPOAb >187 IU/mL, particularly adolescents, should undergo closer monitoring and potential early intervention.

## Data Availability

The datasets generated and analyzed during the current study are not publicly available due to institutional data privacy regulations but are available from the corresponding author on reasonable request.

## Supplementary material

Supplementary Table 1 Age group distribution of excluded and included patients. Note: Data are presented as n (%). Percentages represent column proportions. This table outlines the age distribution across all excluded and included patient subsets to evaluate potential selection or survival bias during the study enrollment process.

Supplementary Table 2 Sensitivity analysis of optimal TPOAb cut-off values across different missing data handling methods. Note: Hazard Ratio (HR) and 95% Confidence Intervals (CI) were derived from univariate Cox proportional hazards regression models.
